# Structural and Environmental Influences Increase the Risk of Sexually Transmitted Infection in a Sample of Female Sex Workers

**DOI:** 10.1097/OLQ.0000000000001400

**Published:** 2021-02-23

**Authors:** Susan G. Sherman, Catherine Tomko, Rebecca Hamilton White, Danielle Friedman Nestadt, Bradley E. Silberzahn, Emily Clouse, Katherine Haney, Noya Galai

**Affiliations:** From the ∗ Department of Health, Behavior, and Society, Johns Hopkins Bloomberg School of Public Health, Baltimore, MD; † Department of Sociology, The University of Texas at Austin, Austin, TX; ‡ Department of Epidemiology, Johns Hopkins Bloomberg School of Public Health, Baltimore, MD

## Abstract

**Background:**

Female sex workers (FSWs) have elevated rates of sexually transmitted infections (STIs) including HIV, yet few studies in the United States have characterized the STI burden in this population.

**Methods:**

Data were derived from the EMERALD study, a structural community-based intervention with FSWs in Baltimore, MD. Participants (n = 385) were recruited through targeted sampling on a mobile van. Prevalent positive chlamydia or gonorrhea infections were determined by biological samples. Multivariable logistic regressions modeled correlates of confirmed positive STI (gonorrhea or chlamydia).

**Results:**

Confirmed STI positive prevalence was 28%, 15% chlamydia and 18% gonorrhea. Approximately two-thirds of the sample (64%) was younger than 40 years, one-third (36%) were Black, and 10% entered sex work in the past year. The sample was characterized by high levels of structural vulnerabilities (e.g., housing instability and food insecurity) and illicit substance use. Female sex workers were more likely to have a positive STI if they had financial dependent(s) (*P* = 0.04), experienced food insecurity at least weekly (*P* = 0.01), entered sex work in the past year (*P* = 0.002), and had 6 or more clients in the past week (*P* = 0.01). Female sex workers were less likely to have a positive STI test result if they were 40 years or older compared with FSW 18 to 29 years old (*P* = 0.02), and marginally (*P* = 0.08) less likely with high (vs. low) social cohesion.

**Conclusions:**

More than a quarter of FSWs had confirmed chlamydia or gonorrhea. In addition to STI risks at the individual level, STIs are driven by structural vulnerabilities. Results point to a number of salient factors to be targeted in STI prevention among FSWs.

## Abstract 2

A study of female sex workers in Baltimore, MD, found a 28% prevalence of gonorrhea or chlamydia, driven by food insecurity, financial dependence, and past-year sex work entry.

Supplemental digital content is available in the text.

Women who exchange sex for money, drugs, or goods face a disproportionate burden of sexually transmitted infections (STIs) including HIV, yet few studies in the United States have characterized the STI burden in this population. Although STI exposure is an occupational hazard, female sex workers (FSWs) are not considered a distinct group in national STI or HIV surveillance data, which effectively minimizes the perceived need for funding prevention and treatment efforts.^1,2^ Research has found a wide range of STI prevalence estimates among FSWs, with variation by location, specific STI, and nature of sex work (e.g., venue vs. street based): chlamydia (4%–15%), gonorrhea (1%–13%), and trichomoniasis (9%–12%).^3–9^ The nature of STI risk among this population is poorly characterized.

The drivers of elevated STI rates among this population are multifaceted. An extant range of structural and environmental factors often impede women's ability to engage in safer sex behaviors, including the criminalization of sex work and other relevant laws (e.g., loitering for street-based FSW), condom coercion, client and other interpersonal violence, police routine and egregious practices, and stigma.^10–14^ The location of sex work is a salient feature of their STI risk profile. Although there are commonalities across some contexts in which sex is sold, the nature of the environmental risk of STIs is dependent on the context (e.g., indoor and street based). Street-based compared with other venues of sex work (e.g., massage parlors and dance clubs) are exposed to higher rates of violence and victimization, less control over their workplace setting, and police abuse.^11,15–17^ Little is known about the collective role of structural vulnerabilities and social factors in positioning FSWs for STI risk in the United States.

Distal structural and socioeconomic factors marginalize certain groups and render them disproportionately susceptible to harm.^18^ These factors manifest as individual-level structural vulnerabilities including financial insecurity, housing instability, food insecurity, and lower levels of education and literacy, which have all been associated with deleterious health outcomes such as STIs, violence, and problematic drug use.^11,19–21^ These vulnerabilities foster internalized and externalized stigma and marginalization.^22,23^ Furthermore, stigmatization and marginalization affect their access to health services and disclosure to providers, including STI testing and treatment.^24–26^ Upon disclosure, FSWs often report experiencing stigma from providers, administrative staff, and other patients, decreasing the likelihood of future health care engagement.

Given the dearth of understanding of STIs among this important yet understudied population in the United States, we examined the individual, interpersonal, and structural correlates of chlamydia and gonorrhea among street-based FSW. Data were derived from one of the first community-level interventions focused on this population, which aimed to reduce harms including HIV, STIs, and problematic drug use among a sample of FSW in Baltimore, MD.

## METHODS

We used baseline data from the EMERALD study, a structural community-based intervention with FSWs in Baltimore, MD. The study protocol including details of methods can be found in Clouse et al. (e.g., E. Clouse et al, unpublished data, 2019).^27^ Briefly, the intervention consists of a drop-in center for guests who do not identify as a man that includes medical care (including reproductive and mental health, medication-assisted treatment for drug use, and primary care) and services such as laundry, lockers for storage, showers, and a safe space to spend time. Using a targeted sampling technique, FSWs were recruited from 10 “zones” of potential sex work activity throughout Baltimore City via mobile van. Six zones were in the intervention area in close proximity to the drop-in center's West Baltimore location, and 4 zones were in the control areas in other areas of the city.^28^

Recruitment was conducted between September 2017 and February 2019. Eligibility criteria included the following: (1) 18 years or older; (2) cisgender woman; (3) sold or traded oral, vaginal, or anal sex “for money or things like food, drugs, or favors in the past 3 months;” (4) picked up clients 3 or more times in the past 3 months; and (5) willing to provide contact information for follow-up visits. Participants completed a survey on a tablet computer using audio computer-assisted self-interview. Participants self-collected samples for STI testing using vaginal swabs from Aptima (Hologic Inc., San Diego, CA); testing took place immediately after survey completion. Participants were paid with a $70 VISA gift card. All study activities were approved by the Johns Hopkins Bloomberg School of Public Health Institutional Review Board. The study was informed by a community advisory board convened from a previous study who advised all aspects of the study. We also have staff with lived experience who informed the data collection methods, surveys, and resources provided to study participants.

### Outcome

After survey completion, participants self-collected biological specimens using Aptima vaginal swabs. Participants were instructed on how to use the swabs and collected their sample in the mobile van bathroom. Samples were stored in sealed tubes until delivered to the laboratory for testing. Specimens were sent for testing at the Baltimore City Health Department (BCHD), which used nucleic acid amplification to test for chlamydia and gonorrhea. Results were available within 2 weeks. The BCHD sent results to the study's field coordinator. Participants were notified of their test results and referred to a BCHD STI clinic for treatment (if positive) during postinterview check-in calls or subsequent field encounters, whichever occurred first. In addition to attempts by EMERALD study staff, FSWs with positive STI results were assigned to a disease intervention specialist at the BCHD who would also attempt to locate and notify the participant of their result. Participants who tested positive for an STI were able to visit a BCHD clinic for free treatment. Referrals to local health and social services—including STI treatment—were offered to all participants upon request and suggested by staff at the end of the survey when staff casually engaged with participants.

The study outcome was defined as a confirmed positive chlamydia or gonorrhea test result. Participants with an indeterminate result were coded as “no confirmed STI positive.”

### Independent Variables

We assessed demographics (e.g., age, race, and education) and structural vulnerabilities including homelessness, hunger (“because there was not enough food to eat”) at least weekly versus less than weekly, arrest, income, and financial dependence. Type, route of administration, and frequency of substance use in the past 6 months were assessed. Binge drinking was defined as having 5 or more drinks in one sitting (from the AUDIT-C^29^). Among women who reported injecting any drug, we asked about frequency of reusing paraphernalia (syringes/needles, cookers, cotton). Participants self-reported past access to and use of health care sources, including past hepatitis C diagnosis and HIV and STI testing. HIV status was assessed using an OraQuick Advanced Rapid HIV-1/2 test kit (Orasure Technologies, Bethlehem, PA).

We measured symptoms of posttraumatic stress disorder using the Posttraumatic Stress Disorder Checklist for Civilians (scores ≥33 indicated clinically significant posttraumatic stress disorder symptoms) and depression using the Patient Health Questionniare-9 (scores >10 indicated the presence of depressive symptoms).^30^^,31s^ We also used a measure of social cohesion previously used in cohorts of FSWs in Brazil, the Dominican Republic, and Tanzania.^32s^ Questions concerned availability of material and emotional support from and closeness among fellow FSWs and were measured on a 4-point Likert scale ranging from strongly agree to strongly disagree. Sense of agency was measured using the Pearlin Mastery Scale.^33s^ Internalized sex work stigma (e.g., “you feel ashamed of sex work” and “you deserve respect as a sex worker”) was also measured on a 4-point scale ranging from strongly agree to strongly disagree.^34s^ We categorized these scores according to tertiles for high, medium, and low scores. We ascertained current health insurance status, having a health care provider, and having been testing for HIV and STI in the past 6 months (outside of the study) with yes/no responses.

Sex work history variables included time spent in sex work (<10 vs. >10 years), age started selling sex, past-year sex work initiation, locations where women met clients, number of clients per week (median split, 1–5 vs. >6), and condomless sex with clients in the past week. We also measured FSW responses to police practices in the past 6 months including rushing negotiations with clients due to police in the area, avoiding carrying condoms, or moving to an unfamiliar area to work to avoid police. Recent client-perpetrated or intimate partner-perpetrated physical or sexual violence was measured using an adapted version of the Revised Conflict Tactic Scale.^35s^ Questions were asked separately of each type of perpetrator.

### Statistical Analysis

χ^2^ Tests were used to determine bivariate between-group differences in independent variables and STI result. Bivariate logistic regression was then used to determine unadjusted correlates of a confirmed STI positive result. Covariates significant at the *P* < 0.20 level were considered for inclusion in the final model in addition to theoretically meaningful variables such as social cohesion, which has been shown as protective against HIV and STI in samples of FSWs.^36s,37s^ We also included recent condomless sex as a potential covariate because it is the most proximate risk factor for STI risk. We used multivariable logistic regression with clustered variance and a manual stepwise selection process to remove covariates from the model. Model fits were considered by comparing Akaike information criterion and Bayesian information criterion values between multivariable models and examining variance inflation factors for multicollinearity. The Hosmer-Lemeshow test was used to assess the goodness-of-fit model.^38s^ Ten women were missing both chlamydia and gonorrhea test results because of a leaked sample or an indeterminate result. We conducted sensitivity analyses by removing these participants from the analysis, rerunning the multivariable model and comparing estimates. All analyses were conducted in Stata/SE 15.1 (College Station, TX).

## RESULTS

The final sample size was n = 385. Confirmed STI prevalence was 28% (confirmed chlamydia prevalence, 18% [95% confidence interval [CI], = 15%–22%]; confirmed gonorrhea prevalence, 15% [95% CI, 12%–20%]). More than half the sample was younger than 40 years (64%), 36% were Black, and 46% finished less than a high school diploma (Table 1). The sample experienced high rates of recent (past 6 months) structural vulnerabilities including homelessness (67%), arrest (27.3%), and food insecurity at least weekly (62%), and high substance use including crack or cocaine (87%), heroin (80%), and injection drug use (58%). Most women had health insurance coverage (85%), and more than half had a usual health care provider (56%). HIV prevalence in the sample was 5%.

**TABLE 1:** Association of Sample Demographics, Structural Vulnerabilities, and Health and Social Characteristics With Prevalent Sexually Transmitted Infection (STI) Among n = 385 Female Sex Workers in Baltimore, MD

	STI Negative/Indeterminate (n = 279; 72.5%), n (column %)	STI Positive(n = 106; 27.5%), n (column %)	Total (n = 385), n (column %)	*P*
**Demographics**				
Age, y				0.002
18–29	53 (19.0)	36 (34.0)	89 (23.1)	
30–39	113 (40.5)	44 (41.5)	157 (40.8)	
40+	113 (40.5)	26 (24.5)	139 (36.1)	
Race				0.32
White	152 (54.5)	66 (62.3)	218 (56.6)	
Black	107 (38.4)	32 (30.2)	139 (36.1)	
Other race	20 (7.2)	8 (7.5)	28 (7.3)	
Relationship status				0.15
Single	198 (71)	83 (78.3)	281 (73)	
In a relationship or married	81 (29)	23 (21.7)	104 (27)	
Education				0.18
Less than a high school diploma	127 (45.5)	50 (47.2)	177 (46.0)	
High school graduate or GED	76 (27.2)	20 (18.9)	96 (24.9)	
At least some college education	76 (27.2)	36 (34)	112 (29.1)	
**Structural vulnerabilities, past 6 mo**				
Homeless	176 (63.1)	81 (76.4)	257 (66.8)	0.01
Someone depends on them financially	127 (45.5)	61 (57.5)	188 (48.8)	0.04
Hungry at least weekly	159 (57.0)	79 (74.5)	238 (61.8)	0.002
Arrest	74 (26.6)	30 (29.1)	104 (27.3)	0.63
**Substance use, past 6 mo**				
Used crack/cocaine	238 (85.3)	96 (90.6)	334 (86.8)	0.17
Used heroin	222 (79.6)	87 (82.1)	309 (80.3)	0.58
Injected heroin and/or cocaine	154 (55.2)	69 (65.1)	223 (57.9)	0.08
Used any drug daily	235 (84.2)	87 (82.1)	322 (83.6)	0.61
Binge drinking*	44 (15.9)	23 (21.9)	67 (17.5)	0.17
**Social factors**				
Social cohesion score*				0.27
Low	86 (31.6)	41 (40.2)	127 (34.0)	
Medium	94 (34.6)	29 (28.4)	123 (32.9)	
High	92 (33.8)	32 (31.4)	124 (33.2)	
**Health and health care characteristics**				
HIV positive^†^	18 (6.5)	2 (1.9)	20 (5.2)	0.08
Have health insurance*	238 (85.9)	84 (80.8)	322 (84.5)	0.22
Currently have usual health care provider*	160 (58)	53 (52)	213 (56.3)	0.3
HIV testing among HIV negative, past 6 months^†^	167 (64.5)	71 (68.3)	238 (65.6)	0.49
STI testing, past 6 mo^†^	175 (63.0)	68 (64.8)	243 (63.5)	0.74

*Less than 5% missing data.

^†^Less than 1% missing data.

Compared with a negative or indeterminate result, FSWs with a confirmed STI positive result were more likely to be between 18 and 29 years old (34.0% vs. 19.0%, *P* = 0.004), homeless in the past 6 months (76.4% vs. 63.1%, *P* = 0.01), have financial dependent(s) (57.5 vs. 45.5, *P* = 0.04), and experience hunger at least weekly (74.5% vs. 57.0%, *P* = 0.002).

Ten percent of the sample began selling sex in the past year (Table 2). Less than half the sample reported condomless sex with clients in the past week (45%). Recent client-perpetrated violence was common including physical violence (32%) and condom coercion (45%). Approximately two-thirds of the sample (64%) had at least one nonpaying intimate partner.

**TABLE 2:** Associations Between Sex Work, Intimate Partner, and Violence Histories and Prevalence Sexually Transmitted Infection (STI) Among Female Sex Workers in Baltimore, MD, Stratified by STI Result (n = 385)

	STI Negative/Indeterminate (n = 279; 72.5%), n (%)	STI Positive (n = 106; 27.5%), n (%)	Total (n = 385), n (%)	*P*
**Sex work history and client violence history**				
Recent (past year) sex work initiation*				
>1 y	257 (93.1)	88 (83.0)	345 (90.3)	0.003
<1 y	19 (6.9)	18 (17.0)	37 (9.7)	
≥6 of clients, past week	144 (51.6)	72 (67.9)	216 (56.1)	0.004
Condomless sex with clients, past week	127 (45.5)	47 (44.3)	174 (45.2)	0.84
Client physical or sexual violence, past 6 mo^†^	116 (41.7)	53 (50.0)	169 (44.0)	0.14
Client condom coercion, past 6 mo*	124 (44.4)	49 (46.2)	173 (44.9)	0.75
**Intimate partner history**				
No. intimate partners, past 6 mo^†^				0.33
None	90 (32.4)	39 (37.1)	129 (33.7)	
1	116 (41.7)	35 (33.3)	151 (39.4)	
≥2	72 (25.9)	31 (29.5)	103 (26.9)	
Condomless sex with intimate partners, past week^‡^	115 (61.2)	35 (53.0)	150 (59.1)	0.25
Intimate partner physical or sexual violence, past 6 months*^‡^	40 (22.4)	17 (26.6)	57 (23.5)	0.49
Intimate partner condom coercion, past 6 mo^‡^	39 (20.7)	18 (27.3)	57 (22.4)	0.27

*Less than 5% missing data.

^†^Less than 1% missing data.

^‡^Of n = 254 with at least 1 intimate partner.

Women who have sold sex for 10 years or longer were less likely to have a confirmed STI positive result (43.4% vs. 64.9%, *P* < 0.001), and women entering sex work in the past year were more likely to have a confirmed STI positive result (17.0% vs. 6.9%, *P* = 0.003). Female sex workers with 6 or more clients in the past week were more likely to have a confirmed STI positive result (67.9% vs. 51.6%, *P* = 0.004).

In unadjusted logistic regression models (Table 3), compared with negative or indeterminate results, FSWs were more likely to have a confirmed positive STI diagnosis if they had at least one financial dependent, experienced food insecurity at least weekly, entered sex work in the past year, and had 6 or more clients in the past week. Female sex workers 30 to 39 years old or 40 years or older were less likely to have a positive STI diagnosis compared with FSWs 18 to 29 years old.

**TABLE 3:** Unadjusted and Adjusted Multivariable Logistic Model of Prevalent STIs in a Sample of Female Sex Workers in Baltimore, MD

	OR (95% CI)	*P*	aOR (95% CI)	*P*
Age (reference: 18–29), y				
30–39	0.57 (0.33–0.99)	0.05	0.69 (0.38–1.26)	0.23
40+	0.34 (0.19–0.62)	<0.001	0.44 (0.23–0.85)	0.02
In a relationship/married (reference: single)	0.68 (0.40–1.15)	0.15	0.55 (0.31–1.00)	0.05
Someone depends on them financially (reference: no)	1.62 (1.03–2.55)	0.04	1.68 (1.02–2.77)	0.04
Food insecurity at least weekly (reference: less than weekly)	2.21 (1.34–3.63)	0.002	1.98 (1.16–3.40)	0.01
Entered sex work in past year (reference: no)	2.77 (1.39–5.51)	0.004	3.43 (1.59–7.41)	0.002
≥6 clients, past week (reference: 1–5)	1.99 (1.24–3.18)	0.004	2.08 (1.24–3.49)	0.01
Social cohesion (reference: low)				
Medium	0.65 (0.37–1.13)	0.13	0.64 (0.35–1.17)	0.15
High	0.73 (0.42–1.26)	0.26	0.59 (0.33–1.06)	0.08

The Hosmer-Lemeshow test showed acceptable model fit (χ^2^ = 214.3, *P* = 0.19). In the multivariable model, FSWs were more likely to have a confirmed positive STI test result if they had financial dependent(s) (adjusted odds ratio [aOR], 1.71; 95% CI, 1.04–2.81), experienced food insecurity at least weekly compared with experiencing food security less frequently (aOR, 2.03; 95% CI, 1.18–3.49), entered sex work in the past year (aOR, 3.63; 95% CI, 1.60–7.49), and had 6 or more clients in the past week (aOR, 1.99; 95% CI, 1.18–3.35). Female sex workers were less likely to have a positive STI test result if they were 40 years or older compared with FSWs 18 to 29 years old (aOR, 0.44; 95% CI, 0.23–0.87) and in a relationship or married compared with single FSWs (aOR, 0.56; 95% CI, 0.31–1.01). When controlling for other variables, compared with the unadjusted model, there was virtually no change in estimates among FSWs with medium social cohesion compared with low, but there was a trend toward a significant (*P* = 0.08) protective effect on STI diagnosis for FSWs with high social cohesion compared with low (aOR, 0.59; 95% CI, 0.33–1.09). Point estimates were comparable in the sensitivity analyses, and interpretation of results did not change from the final model.

## DISCUSSION

The study found that more than a quarter of our sample of FSWs in Baltimore, MD, had prevalent chlamydia or gonorrhea. This high prevalence echoes findings from other research including previous work of ours in Baltimore and underscore the sense of urgency for a timely response. Sexually transmitted infections are a unique occupational hazard largely driven by structural vulnerability and potentiated by the criminalization of sex work.^3–9^^,39s,40s^ Structural and occupational factors elevated women's risk of a prevalent STI. Being hungry at least weekly, having someone financially dependent on the participant, entering sex work within the past year, and having a high number of weekly clients conferred elevated odds of an STI infection. Being older, along with 2 measures of social connectedness—being in an intimate relationship and a high level of social cohesion—trended toward protection from STIs. Interventions with FSWs should approach STI prevention from a multilevel perspective, focusing on measures of social connectedness as well as structural and occupational factors.

Food security is a basic human right that is often not afforded to marginalized populations. Housing is another human right, yet in our and others' work, homelessness is consistently associated with elevated risks of sexual and physical violence, HIV, and STIs.^41s–44s^ Less research has examined food insecurity. We and others in Vancouver have found a significant association between HIV infection, increased HIV risk, and food insecurity.^43s–45s^ Food insecurity is also associated with an increased risk of intimate partner and client-perpetrated violence, which in turn are strongly tied to poor mental health and substance use.^47s–47s^ The relationship between food insecurity and violence is often driven by the pressure of few economic resources and exertion of control over women by partners.^43s,45s,48s^

Additional economic strains such as caring for financial dependents adds to the financial stressors structurally vulnerable FSWs experience; indeed, we found an independent association between having financial dependents and prevalent STI. Lack of economic opportunities other than sex work is one of the reasons women enter the field, and demands for income and food security create pressure to engage in riskier acts with clients for higher pay, such as forgoing condom use.^43s,49s–51s^ It is important to note that this analysis is limited by cross-sectional data, and we therefore cannot determine the temporality or causality of economic strains or food insecurity on prevalent STIs. Our results, however, add to the growing body of literature on the importance of addressing social and structural determinants of health such as food and financial security to more fully stem the HIV epidemic and its associated risk factors in FSWs.

We found that younger (18–29 years) FSWs were more likely to test positive for an STI compared with older (40+ years) FSWs, which is supported by similar to the distribution of chlamydia and gonorrhea in the general population. We also found that sex work characteristics conferred STI risk including recent sex work initiation and low social cohesion with other FSWs. Recent sex work initiation was associated with a more than 3-fold increase in the risk of STI diagnosis even when controlling for number of clients, a result also found in a previous analysis of STI diagnoses in Baltimore-based FSWs.^3^ Women recently entering sex work may have current life circumstances or lack of experience with clients that are not captured by our survey and which render them more vulnerable to STIs. High social cohesion was marginally (*P* = 0.08) associated with prevalent STIs in the adjusted model. Although this was only a marginally significant finding, we feel it is worthy of discussion given low levels of and potentially positive effect of being socially connected. Our measure of social cohesion asks about connections between FSWs that directly help women learn about specific clients, seek out health care, and broadly bolster their perceived ability to practice safer sex—all strongly protective against STIs. Fostering this sense of connection can be crucial to promoting health and safety among FSWs, particularly new initiates. Globally, social cohesion is consistently found to be protective against a host of poor health outcomes for FSWs including consistent condom use,^32s,37s,52s,53s^ better psychosocial outcomes (e.g., reduced suicidality and fewer experiences of social discrimination), and lower odds of client condom refusal.^36s,52s,54s^ There may also be social STI drivers that are not captured by our study including the size of the FSWs and paying client populations and the extent to which they are interlinked. Studies that attempt to capture the structure of sexual networks, including the clients, are needed to better understand this potentially key aspect of risk.

We were surprised with the lack of significant relationship between several variables and having an STI, the first of which is use of either an illicit opioid or stimulant. But perhaps the high prevalence of illicit drug use and the important of other variables overshadowed the impact of drug use. Furthermore, unlike our previous analysis of predictors of incident STIs, which found health care access to be a significant factor, we did not find that in the current analysis.^3^ Lastly, no measure of violence was significant in our multivariable model, a finding that departs from the aforementioned prior analysis of STI incidence.^3^

This study adds to the small but growing body of literature showing the importance of structural and environmental factors on FSWs' STI risk. Although seemingly disparate, attending to hunger, a sign of severe economic hardship, could reduce the necessity to engage in sexual behaviors that place women at risk for STIs. Furthermore, national STI surveillance among this population would provide a deeper understanding of the nature of STI prevalence and its correlates over time by region, ethnicity, and a number of important factors. The lack of surveillance leads to a lack of funding and intervention focus among FSWs in the United States. Broadly speaking, FSWs' risk is structured largely in a context of criminalized employment, which amplifies the impact of such structural factors as food insecurity and sex work–related factors such as length of sex work or the number of clients. Sex work decriminalization is an essential component of improving the health and human rights of all sex workers, including STI and HIV prevention. As such, it is recommended by international organizations including UNAIDS, Amnesty International, and the World Health Organization.^55s–57s^ Although upstream from the study's main findings, decriminalization would deflate some of the drivers of STIs among this population, reducing both the role of existing structural vulnerabilities (e.g., food insecurity) and what should simply be occupational descriptions (e.g., length of time at work and number of clients). Future research and clinical efforts that aim to decrease STI prevalence in this population, particularly with structurally vulnerable FSW, must address salient factors at multiple socioecological levels to holistically intervene on STI risk.

## Supplementary Material

SUPPLEMENTARY MATERIAL

## Appendix

For further references, please see “Supplemental References,” http://links.lww.com/OLQ/A643.
