# Supplementary material for: Structural and Environmental Influences Increase the Risk of Sexually Transmitted Infection in a Sample of Female Sex Workers
Source: Sex Transm Dis. 2021 Feb 23;48(9):648–53. doi: 10.1097/OLQ.0000000000001400 (PMC8360669; doi:10.1097/OLQ.0000000000001400)
Supplement: SUPPLEMENTARY MATERIAL [file std-48-0648-s001.docx]

31s. Weathers FW LB, Herman D, Huska J, Keane T. *The PTSD checklist-civilian version (PCL-C).* Boston, MA: National Center for PTSD; 1994.

32s. Lippman SA, Donini A, Diaz J, Chinaglia M, Reingold A, Kerrigan D. Social-environmental factors and protective sexual behavior among sex workers: the Encontros intervention in Brazil. *Am J Public Health.* 2010;100 Suppl 1:S216-223.

33s. Pearlin LI, Schooler C. The structure of coping. *Journal of health and social behavior.* 1978:2-21.

34s. Tomko C, Nestadt DF, Rouhani S, et al. Confirmatory Factor Analysis and Construct Validity  of  the Internalized  Sex  Work Stigma Scale among a Cohort  of  Cisgender Female  Sex  Workers in Baltimore, Maryland, United States. *Journal of Sex Research.* 2020;Accepted for publication.

35s. Straus MA, Douglas EM. A short form of the Revised Conflict Tactics Scales, and typologies for severity and mutuality. *Violence Vict.* 2004;19(5):507-520.

36s. Argento E, Duff P, Bingham B, et al. Social Cohesion Among Sex Workers and Client Condom Refusal in a Canadian Setting: Implications for Structural and Community-Led Interventions. *AIDS Behav.* 2016;20(6):1275-1283.

37s. Carrasco MA, Barrington C, Perez M, Donastorg Y, Kerrigan D. Social cohesion, condom use, and sexually transmitted infections among female sex workers living with HIV in the Dominican Republic. *Int J STD AIDS.* 2019;30(1):64-71.

38s. Hosmer DW, Lemesbow S. Goodness of fit tests for the multiple logistic regression model. *Communications in statistics-Theory and Methods.* 1980;9(10):1043-1069.

39s. Decker MR, Wirtz AL, Baral SD, et al. Injection drug use, sexual risk, violence and STI/HIV among Moscow female sex workers. *Sex Transm Infect.* 2012;88(4):278-283.

40s. Shahmanesh M, Cowan F, Wayal S, Copas A, Patel V, Mabey D. The burden and determinants of HIV and sexually transmitted infections in a population-based sample of female sex workers in Goa, India. *Sex Transm Infect.* 2009;85(1):50-59.

41s. Duff P, Deering K, Gibson K, Tyndall M, Shannon K. Homelessness among a cohort of women in street-based sex work: the need for safer environment interventions. *BMC Public Health.* 2011;11:643.

42s. Duff P, Goldenberg S, Deering K, et al. Barriers to viral suppression among female sex workers: role of structural and intimate partner dynamics. *Journal of acquired immune deficiency syndromes (1999).* 2016;73(1):83.

43s. Lim S, Park JN, Kerrigan DL, Sherman SG. Severe Food Insecurity, Gender-Based Violence, Homelessness, and HIV Risk among Street-based Female Sex Workers in Baltimore, Maryland. *AIDS Behav.* 2019;23(11):3058-3063.

44s. Sherman SG, Park JN, Galai N, et al. Drivers of HIV infection among cisgender and transgender female sex worker populations in Baltimore city: Results from the SAPPHIRE study. *J Acquir Immune Defic Syndr.* 2019.

45s. Barreto D, Shannon K, Taylor C, et al. Food Insecurity Increases HIV Risk Among Young Sex Workers in Metro Vancouver, Canada. *AIDS and Behavior.* 2017;21(3):734-744.

46s. Miller CL, Bangsberg DR, Tuller DM, et al. Food insecurity and sexual risk in an HIV endemic community in Uganda. *AIDS Behav.* 2011;15(7):1512-1519.

47s. Ricks JL, Cochran SD, Arah OA, Williams JK, Seeman TE. Food insecurity and intimate partner violence against women: results from the California Women's Health Survey. *Public Health Nutr.* 2016;19(5):914-923.

48s. Whittle HJ, Leddy AM, Shieh J, et al. Precarity and health: Theorizing the intersection of multiple material-need insecurities, stigma, and illness among women in the United States. *Soc Sci Med.* 2020;245:112683.

49s. Deering KN, Lyons T, Feng CX, et al. Client demands for unsafe sex: the socioeconomic risk environment for HIV among street and off-street sex workers. *J Acquir Immune Defic Syndr.* 2013;63(4):522-531.

50s. McCarthy B, Hagan J. Surviving on the street: The experiences of homeless youth. *Journal of Adolescent Research.* 1992;7(4):412-430.

51s. Sanders T. A continuum of risk? The management of health, physical and emotional risks by female sex workers. *Sociology of health & illness.* 2004;26(5):557-574.

52s. Fonner VA, Kerrigan D, Mnisi Z, Ketende S, Kennedy CE, Baral S. Social cohesion, social participation, and HIV related risk among female sex workers in Swaziland. *PloS one.* 2014;9(1):e87527.

53s. Kuhlmann AS, Galavotti C, Hastings P, Narayanan P, Saggurti N. Investing in communities: evaluating the added value of community mobilization on HIV prevention outcomes among FSWs in India. *AIDS Behav.* 2014;18(4):752-766.

54s. Argento E, Strathdee SA, Shoveller JA, Braschel M, Shannon K. Correlates of Suicidality Among A Community-Based Cohort of Women Sex Workers: The Protective Effect of Social Cohesion. *J Interpers Violence.* 2019:886260519870167.

55s. World Health Organization. Prevention and treatment of HIV and other sexually transmitted infections for sex workers in low-and middle-income countries: recommendations for a public health approach. 2012.

56s. Amnesty International. Policy on state obligation to respect, uphold and fulfil the human rights of sex workers. <https://www.amnesty.org/en/documents/pol30/4062/2016/en/> Published 2016. Updated May 26, 2016. Accessed January 14, 2021.

57s. Joint United Nations Programme on HIV/AIDS. UNAIDS guidance note on HIV and sex work. *Geneva: UNAIDS.* 2012.
